# Risk‐Profile Based Monitoring Intervals for Multivariate Longitudinal Biomarker Measurements and Competing Events With Applications in Stable Heart Failure

**DOI:** 10.1002/sim.70088

**Published:** 2025-05-16

**Authors:** Teun B. Petersen, Eric Boersma, Isabella Kardys, Dimitris Rizopoulos

**Affiliations:** ^1^ Department of Biostatistics Erasmus MC University Medical Center Rotterdam the Netherlands; ^2^ Department of Epidemiology Erasmus MC University Medical Center Rotterdam the Netherlands; ^3^ Department of Cardiology, Thorax Center, Cardiovascular Institute Erasmus MC University Medical Center Rotterdam the Netherlands

**Keywords:** decision‐making, dynamic risk prediction, heart failure, joint models for longitudinal and time‐to‐event data, personalized medicine

## Abstract

Patient monitoring is routinely used to detect disease aggravation in many chronic conditions. We propose an adaptive scheduling strategy based on dynamic individual risk predictions that can improve the efficiency of monitoring programs that incorporate multiple longitudinal measurements and competing events. It is motivated by stable chronic heart failure (CHF) patients who are periodically seen to assess the risk of disease aggravation based on multiple patient characteristics and circulating marker protein levels such as NT‐proBNP and troponin. We assess the performance of the adaptive strategy versus fixed schedule alternatives using a simulation study based on the Bio‐SHiFT study, a cohort of stable CHF patients.

## 1 Introduction

Patient monitoring has become an invaluable tool in clinical practice for many chronic conditions susceptible to adverse events. Here, patients are periodically seen for informative biomarker measurements or disease progression screenings, with the aim of catching disease aggravation and preventing events via invasive procedures or altered medical treatment. The time between monitoring visits is generally fixed, and literature on optimizing this fixed interval is often limited [1]. However, studies from our group have indicated that the efficiency and efficacy of monitoring can be improved by incorporating patient‐specific information into the decision when to schedule the next visit [2, 3, 4]. Until now, most of these studies focused on one repeatedly measured biomarker and one survival outcome, while in practice, disease progress is captured by multiple biomarkers reflecting various pathophysiological pathways, and competing risks can exist with varying clinical consequences. In this article, we apply these concepts to multiple longitudinal outcomes and competing risks scenarios to make application in clinical settings more feasible.

The motivation for this extension stems from stable chronic heart failure (CHF) patients, who are often monitored for disease aggravation resulting in hospitalization and other adverse cardiovascular events. Heart failure (HF) is a heterogeneous condition where many underlying processes can influence disease progression [5]. Two important prognostic biomarkers that are often considered during monitoring visits are serum protein levels of NT‐proBNP and troponin, which indicate stretching of heart ventricles in response to increased blood volume and heart muscle damage, respectively. Elevated levels of either marker predict increased hazard for various adverse cardiovascular events [6].

The monitoring scenario for this study is as follows: Consider that we have a patient i followed up to a time point t, which has given us some biomarker measurements until that time. At time t, a clinician is interested in the risk of a specific event occurring before the horizon time t+Δ; if the risk exceeds a certain clinically motivated threshold κ, the monitoring process is interrupted to adjust medical therapy. If this cumulative risk is lower than κ, the patient remains to be followed, and an appointment is scheduled for a new measurement of the biomarkers at time $t+u_{i}(t)$. In this study, we focus on optimizing the way to choose $u_{i}(t)$ depending on the patient's history observed at time t. Frequent biomarker measurements can be burdensome for the patient and the healthcare system, while appointments scheduled too sporadically could endanger the patient's chances of timely medication adjustments. To address this challenge, we present an adaptive scheduling strategy incorporating dynamic risk predictions and illustrate its benefits in patient monitoring using a simulation study based on a cohort of stable CHF patients included in the Bio‐SHiFT study conducted in Rotterdam and Alkmaar, the Netherlands.

The remainder of the paper is structured as follows: In Section 2, we give an overview of the theory and framework behind obtaining dynamic personalized risk predictions using joint models for longitudinal and time‐to‐event data and how to use them to optimize monitoring intervals. Section 3 presents the simulation methods used to evaluate the performance of the adaptive scheduling strategy in a population of stable CHF patients. Finally, Section 4 discusses the results of this simulation study.

## 2 Optimizing Monitoring Schedules Using Dynamic Risk Predictions

In this section, we will first describe ways to estimate dynamic personalized risk estimates for individual patients at a certain time before discussing how these estimates can be used to pick more efficient schedules.

### 2.1 Joint Model for Longitudinal and Time‐To‐Event Data

Let $T_{ik}^{*}$ denote the true failure time for patient i∈{1,…,n} and competing risk k∈{1,…,K}. $T_{i}=\min(C_{i},T_{i1},\ldots ,T_{iK})$ denotes the observed failure time for patient i, where $C_{i}$ is the time of censoring. $\delta_{i}\in \{0,\ldots ,K\}$ indicates which of the K events has taken place or, when it is 0, whether the patient was censored before any event occurred. Let $y_{i1},\ldots ,y_{iP}$ be the P longitudinal response vectors of patient i. The length of these vectors and their times of measurement are allowed to vary among the longitudinal responses. Biomarker trajectories are described using generalized linear mixed‐effect models, specified as:

$$g\left\{E(y_{ip}|b_{ip})\right\}=\eta_{ip}(t)=x_{ip}^{\prime }\beta_{p}+z_{ip}^{\prime }b_{ip}$$

where the distribution of the pth (p∈{1,…,P}) longitudinal outcome conditional on its random effects $\left(y_{ip}|b_{ip}\right)$ is assumed to be a member of the exponential family with a mean conditional on the random effects. g(x) is a link function, t is the time, $x_{ip}^{\prime }$ is the transpose of a design vector for the fixed effect regression coefficients β, and $z_{ip}$ is a design vector for the random effect coefficients $b_{ip}$. The joint distribution of the random effects for all longitudinal outcomes $b_{i}=(b_{i1}^{\prime },\ldots ,b_{iP}^{\prime })$ is assumed to be multivariate normal with mean 0 and variance‐covariance matrix D.

The cause‐specific hazard functions of the survival model have the form

$$\begin{array}{ll} h_{ki}(t|H_{i},w_{ki}) & =h_{k}^{0}(t)\exp\left\{\gamma_{k}^{\prime }w_{ki}+\alpha_{k}^{\prime }f_{k}(H_{i}(t),b_{i})\right\},\\ H_{i}(t) & =\{\eta_{i1}(s),\ldots ,\eta_{iP}(s)|0\leq s\leq t\} \end{array}$$

where $h_{k}^{0}$ is the baseline hazard, $w_{ki}$ and $\gamma_{k}$ are the baseline covariates and coefficients, and $\alpha_{k}$ is a vector of coefficients expressing the strength of association between each function $f_{kj}(H_{i}(t),b_{i})$ and the outcomes. The longitudinal response is incorporated into the survival model by making the cause‐specific hazard functions dependent on one or more functions of the patient‐specific linear predictors up to time point t or the random effect coefficients $b_{i}$. These functions $f_{kj}(H_{i}(t),b_{i})$ can range from simply the value of a linear predictor ($f_{kj}(H_{i}(t),b_{i})=\eta_{ip}(t)$) to its derivative ($f_{kj}(H_{i}(t),b_{i})=\frac{d\eta_{ip}(t)}{dt}$) or exposure/area under the curve ($f_{kj}(H_{i}(t),b_{i})=\int_{0}^{t}\eta_{ip}(s)ds$). The model is estimated via a Bayesian approach using the *JMbayes2* R‐package [7].

### 2.2 Dynamic Predictions of Cumulative Incidence

Dynamic predictions for cumulative incidence have been studied extensively via landmarking [8] and joint models for longitudinal and time‐to‐event data [9]. Here we are interested in estimating $\pi_{ik}(t,s)=\mathrm{Pr}(T_{ik}^{*}<s|\cup_{k=1}^{K}T_{ik}^{*}>t,{\tilde{Y}}_{i}(t),D_{n})$, the probability of the kth event occurring before some time s given all measurements available until time t, and the fact that no event occurred before time t, where ${\tilde{Y}}_{i}(t)$ is the set of all longitudinal measurements of patient i up to time t, and $D_{n}$ is the dataset used to fit the model. Under the Bayesian formulation of the joint model, $\pi_{ik}(t,s)$ can be estimated using posterior predictive distribution

$$\begin{array}{ll} \pi_{ik}(t,s) & =\mathrm{Pr}(T_{ik}^{*}<s|\cup_{k=1}^{K}T_{ik}^{*}>t,{\tilde{Y}}_{i}(t),D_{n})\\ & =\int \mathrm{Pr}(T_{ik}^{*}<s|\cup_{k=1}^{K}T_{ik}^{*}>t,{\tilde{Y}}_{i}(t);\theta )\mathrm{Pr}(\theta |D_{n})d\theta \end{array}$$

where

$$\begin{array}{ll} & \;\mathrm{Pr}(T_{ik}^{*}<s|\cup_{k=1}^{K}T_{ik}^{*}>t,{\tilde{Y}}_{i}(t);\theta )\\ & =\int \mathrm{Pr}(T_{ik}^{*}<s|\cup_{k=1}^{K}T_{ik}^{*}>t,{\tilde{Y}}_{i}(t),b_{i};\theta )\\ & \;\times \mathrm{Pr}(b_{i}|\cup_{k=1}^{K}T_{ik}^{*}>t,{\tilde{Y}}_{i}(t);\theta )db\\ & =\int \frac{\mathrm{Pr}(T_{ik}^{*}<s,\cup_{k=1}^{K}T_{ik}^{*}>t|b_{i};\theta )}{\mathrm{Pr}(\cup_{k=1}^{K}T_{ik}^{*}>t|b_{i};\theta )}\\ & \;\times \mathrm{Pr}(b_{i}|\cup_{k=1}^{K}T_{ik}^{*}>t,{\tilde{Y}}_{i}(t);\theta )db\\ & =\int \frac{CIF(t,s)}{S(t)}\mathrm{Pr}(b_{i}|\cup_{k=1}^{K}T_{ik}^{*}>t,{\tilde{Y}}_{i}(t);\theta )db \end{array}$$

As such, $\pi_{ik}(t,s)$ can be estimated using a Monte Carlo scheme [9].

### 2.3 Optimization of Biomarker Measurement Schedules

Consider a patient at time t in a situation where we are interested in only one of the K events. If the probability of this event occurring within Δ time units exceeds a threshold κ ($\pi_{ik}(t,t+\Delta )\geq \kappa$), patient monitoring is interrupted by the clinician to adjust medical therapy in an effort to prevent the event. If not, a new biomarker measurement is scheduled at time $t+u_{i}(t)$. We assume that all biomarkers are measured at the same time, as is standard in the context of the serum proteins measured in our motivating dataset. To improve the monitoring process, we would like to optimize two aspects of $u_{i}(t)$ via a novel scheduling strategy. First, the accuracy of identifying the need for therapy adjustment, and second, minimizing the number of necessary measurements.

**FIGURE 1:**
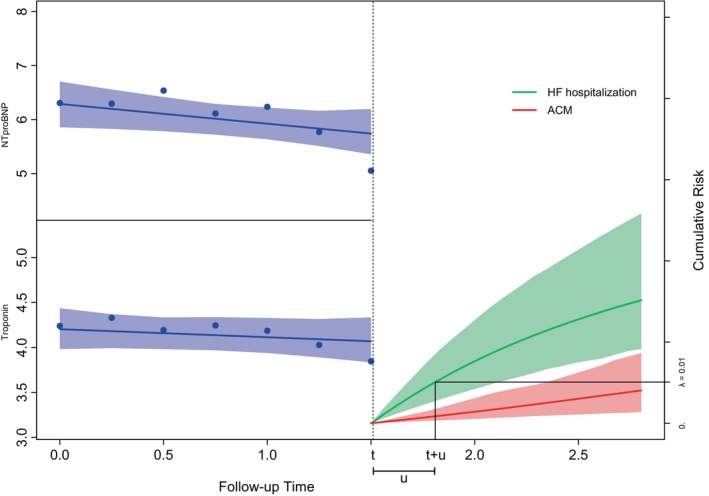
Example of the two longitudinal responses measured for a patient until time point 1.5 and cumulative incidence functions for both competing hazards from this time. The optimal time point for a new screening is highlighted at the point where the cumulative incidence of HF hospitalization exceeds λ=1%.

We assume that patients with a higher risk of the adverse event should be seen more frequently than stable patients with lower risks, as they have a higher probability of needing therapy adjustment in the near future. We can use this notion in a scheduling strategy by making the time until the next measurement inversely proportional to the risk of an event of interest occurring. This can be achieved by limiting the estimated cumulative risk before the next measurement. This risk limit λ can be chosen in agreement between patients and clinicians using a decision curve to balance net benefits and risk [10]. In the special case where λ=κ, monitoring is interrupted when the optimal interval $u_{\text{opt}}$ drops below Δ. As $\pi_{ik}(t,s)$ is a strictly increasing function in s, we can write this solution as follows:

(2.1) $$u_{\text{opt},i}(t)=\{u\in (0,u_{\max})|{\hat{\pi }}_{ik}(t,t+u)=\lambda \}$$

where $u_{\max}$ is a potential upper limit on the time until the next measurement, and ${\hat{\pi }}_{ik}(t,t+u)$ is the Monte Carlo estimate of $\pi_{ik}(t,t+u)$. This strategy would ensure longer time intervals between measurements when the hazard is low, improving efficiency, and reduce the time between measurements when the hazard is high, which could allow for more accurate predictions of the optimal time of interruption. This strategy is illustrated in Figure 1. It can be adjusted to be more conservative by taking the upper 95% confidence interval limit of the risk estimate as follows:

(2.2) $$u_{\text{opt},i}(t)=\{u\in (0,u_{\max})|\mathrm{Pr}(\pi_{ik}(t,t+u)\leq \lambda )=0.05/2\}$$

to be more sure that the real risk of an event does not exceed λ. The strategy of (2.1) can be generalized to work with multiple types of therapy adjustment corresponding to varying endpoints by estimating an optimal $u_{opt,k}$ for every endpoint and taking the minimum value such that

$$\begin{array}{ll} \forall k & \in \{1,\ldots ,K\},\\ u_{\text{opt},i,k}(t) & =\{u_{k}\in (0,u_{\max,k})|{\hat{\pi }}_{ik}(t,t+u_{k})=\lambda_{k}\},\\ u_{\text{opt},i}(t) & =\min(u_{\text{opt},1}(t),\ldots ,u_{\text{opt},K}(t)) \end{array}$$

where $u_{\max,k}$ and $\lambda_{k}$ are endpoint‐specific versions of $u_{\max}$ and λ.

## 3 Bio‐SHiFT Data Analysis

We return to the motivating example of clinical monitoring in stable CHF patients. The performance of the risk‐profile based strategy cannot be evaluated using real retrospective data, as all measurement timings are already fixed. Therefore, to evaluate the performance of our proposed method for selecting the time intervals to collect longitudinal measurements, we will resort to a simulation study presented in the next section. The parameters of our simulation will be based on the Serial Biomarker Measurements and New Echocardiographic Techniques in Chronic Heart Failure Patients Result in Tailored Prediction of Prognosis (Bio‐SHiFT) study, which was conducted in the Erasmus MC, Rotterdam, and Northwest Clinics, Alkmaar, Netherlands. This was a prospective cohort study of stable CHF patients [11]. Patients were recruited during regular outpatient visits and included if they were 18 years or older, able to understand and sign the informed consent form, and if they were not diagnosed with CHF, or hospitalized for HF, less than 3 months before inclusion. Information about the patients was recorded at baseline and at predefined follow‐up visits, which were scheduled every 3 (± 1) months. These visits included short medical examinations, collection of blood samples, and documentation of adverse cardiovascular events since the last visit. A total of 398 CHF patients were enrolled between August 2011 and January 2018. This investigation concerns the 381 patients with reduced ejection fraction (HFrEF) and trimonthly measurements of NT‐proBNP and troponin T. The study was approved by the medical ethics committee of the Erasmus Medical Center in Rotterdam and complied with the Declaration of Helsinki. All included patients provided written informed consent.

For the survival part of our simulation, we considered two endpoints: HF hospitalization (90/381) and a competing outcome of all‐cause mortality (14/381). For the longitudinal model, we take two circulating proteomic biomarkers commonly associated with HF: n‐terminal prohormone of brain natriuretic peptide (NT‐proBNP) and cardiac muscle troponin T (TNNT2) [6]. Both longitudinal outcomes are log‐transformed and modeled using random intercepts and slopes. The functional form of the cause‐specific hazard functions was chosen such that model fit, as reflected by the WAIC, is optimized. Measures of model fit for different combinations of value, slope, and area under the curve are illustrated in Table 1. As such, they were specified as follows:

(3.1) $$\begin{array}{ll} h_{ki}(t) & =h_{k}^{0}(t)\exp(\gamma_{k}{\text{maggic}}_{i}+\alpha_{k,1}\eta_{i,\text{NT‐proBNP}}(t)\\ & \;+\alpha_{k,2}\eta_{i,\text{TNNT2}}(t)) \end{array}$$

using the linear predictor of the two longitudinal outcomes and the Meta‐Analysis Global Group in Chronic Heart Failure Risk Score (MAGGIC) [12], which corrects for clinical characteristics at baseline. The MAGGIC risk score incorporates age; sex; diabetes mellitus, COPD, and smoking status; HF duration; NYHA class; beta‐blocker, ACE‐inhibitor and ARB use; systolic blood pressure; body mass index; serum creatinine; and ejection fraction.

**TABLE 1:** Model fit of the joint model given functional form of the hazard functions in order of minimal WAIC.

NT‐proBNP	Troponin T	WAIC	DIC	LPML
Value	Value	7313.60	7433.51	−3665.99
Value + area	Value + area	7315.77	7433.48	−3670.25
Value + area	Value	7318.23	7433.93	−3672.21
Value	Area	7318.37	7437.48	−3668.98
Value + area	Area	7318.84	7434.75	−3671.12
Value + slope	Area	7320.33	7437.20	−3680.68
Area	Value + area	7320.35	7436.11	−3674.30
Value + slope	Value	7320.80	7437.64	−3676.01
Value + slope	Value + area	7322.99	7438.60	−3682.49
Value	Value + area	7323.17	7436.34	−3675.98
Area	Area	7323.32	7443.99	−3671.27
Area	Value	7323.95	7443.09	−3671.49
Value + area	Value + slope	7325.89	7440.88	−3706.44
Value	Value + slope	7337.06	7445.33	−3706.91
Value + area	Slope	7338.65	7453.38	−3688.14
Area	Value + slope	7342.69	7446.64	−3726.90
Value + slope	Value + slope	7354.54	7454.12	−3734.56
Slope	Value	7358.87	7479.07	−3694.63
Slope	Area	7361.16	7482.10	−3691.87
Area	Slope	7364.38	7464.63	−3781.10
Value	Slope	7385.17	7463.87	−3956.73
Slope	Value + slope	7412.27	7492.10	−3889.01
Slope	Value + area	7413.50	7479.68	−3985.72
Slope	Slope	7446.46	7542.67	−3754.25
Value + slope	Slope	7670.18	7470.91	−4236.54

Table 2 displays the model coefficients estimated using the *JMBayes2* R‐package. Both NT‐proBNP and troponin T are significantly associated with HF hospitalization. The association between both NT‐proBNP and troponin T and all‐cause mortality does not differ significantly from their association with HF hospitalization. Prediction accuracy for HF hospitalization was assessed using 5‐fold cross‐validated time‐varying AUC and Brier scores adapted to the competing risk setting [13]. Assessing the one‐year ahead predictions one year after baseline, we find a mean AUC of 0.81 (SD 0.04 across folds) and a mean brier score of 0.13 (SD 0.02 across folds).

**TABLE 2:** Parameters of the joint model fitted on the Bio‐SHiFT data.

Longitudinal outcome	Mean	StDev	2.50%	97.50%	*p*	Rhat
log[Troponin T (ng/L)]						
(Intercept)	2.8788	0.0503	2.7805	2.9778	0.0000	0.9999
Time	0.0537	0.0126	0.0294	0.0787	0.0000	1.0018
Sigma	0.2114	0.0036	0.2046	0.2187	0.0000	1.0007
log[NT‐proBNP (pmol/L)]						
(Intercept)	4.6624	0.0778	4.5081	4.8158	0.0000	1.0000
Time	0.0472	0.0257	−0.0024	0.0981	0.0612	1.0012
Sigma	0.4105	0.0068	0.3973	0.4241	0.0000	1.0004
Survival outcome						
maggic	0.0112	0.0296	−0.0473	0.0672	0.7067	1.0023
maggic:strata(strata)2	−0.0200	0.0538	−0.1208	0.0935	0.6867	1.0310
Value(Troponin)	0.5080	0.2137	0.0880	0.9205	0.0172	1.0024
Value(Troponin):strata2	−0.0314	0.5063	−1.0734	0.8830	0.9722	1.0338
Value(NTproBNP)	0.9957	0.1638	0.6785	1.3197	0.0000	1.0153
Value(NTproBNP):strata2	−0.2787	0.3458	−0.9606	0.3807	0.4191	1.0996

*Note:* strata1 = HF hospitalization; strata2 = all‐cause mortality.

## 4 Performance of Risk‐Profile Based Monitoring Intervals

In this section, we describe the simulation scheme used to quantify the efficiency and accuracy of the described scheduling strategy and report the results.

### 4.1 Simulation

Every simulation iteration goes as follows: First, we generate a new dataset of true survival times and biomarker trajectories for 431 patients using the estimated parameters from the joint model fitted on the Bio‐SHiFT data. The baseline hazard function $h_{k}^{0}(t)$ is assumed to be Weibull‐distributed with scale and shape parameters calibrated such that the simulations match the observed data. The dataset is simulated as follows:

1. Draw random effects of the longitudinal outcomes $b_{i}∼N(0,D)$, which can be used to generate “observed” biomarker measurements at any time by adding a measurement error $\epsilon_{ip}∼N(0,\sigma_{p}^{2})$ to the value of $\eta_{ip}(t)$, and baseline characteristic $maggic_{i}∼N(\mu_{maggic},\sigma_{maggic}^{2})$ for every simulated patient.
2. Estimate the hazard functions for each type of event as described in (3.1) and the corresponding cumulative overall hazard function:
$$H_{i}(t)=\sum_{k=1}^{K}\int_{0}^{t}h_{ki}(t|H_{i},w_{ki})$$
3. Estimate the cumulative density function for overall survival: $F_{i}(t)=1-\exp(-H_{i}(t))$ and draw an overall event time using inverse transform sampling (draw a number from a continuous uniform distribution between 0 and 1, and use this as input in the inverse of the CDF to determine $T^{*}$).
4. Censor patients at year 20 if $T^{*}\geq 20$; otherwise, determine event type given the overall event time using a binomial trial with probabilities proportional to the hazard at time point $T^{*}$ [14].

The simulated patients are divided into two groups, one for training a new joint model consisting of 381 patients (the size of the Bio‐SHiFT cohort) and one test group of 50 patients to assess the performance of different scheduling strategies. For the patients in the training group, we simulate biomarker measurements at fixed quarterly intervals and use them to fit a joint model to make dynamic risk predictions in the test group. For the test group, we simulate biomarker measurements at three regular intervals, annually, semi‐annually and quarterly, and at optimized intervals using our strategies previously described in (2.1) and (2.2) with λ=1% and $u_{\max}=\Delta =1$ year, until we find that the risk of the event occurring within Δ=1 year exceeds κ=10%, or an endpoint has occurred before that time. To improve initial predictions, the strategies include a warm‐up period for the first half a year with quarterly equally spaced measurements, where no risk predictions are made. This whole process is repeated for 500 simulation iterations.

The performance of the strategies is compared using three criteria. First, the mean number of measurements taken per patient and per patient year after the warm‐up period. Second, the accuracy of identifying the need for interruption for therapy adjustment. We define the optimal time of interruption as the first time that the true cumulative incidence ($CIF_{k}(a,b)=\int_{a}^{b}h_{k}(u)S(u)du$) of the patient within Δ=1 years exceeds κ=10% ($t_{optimal}=\min(\{t\in [0,\infty )|CIF_{k}(t,t+\Delta )=\kappa \})$. Patients where $t_{optimal}$ falls before an endpoint are deemed to be in need of interruption, and this is identified if the strategy interrupts the monitoring process for these patients before the endpoint occurs. Finally, if monitoring is interrupted, how close does this time of interruption resemble the optimal time of interruption. We quantified this using the root mean squared error (RMSE) between the realized and optimal interruption times.

To examine how robust the strategy is to model misspecification, the simulations are repeated in a setting where the normality of the residuals in the longitudinal part and the proportional hazards assumption are violated while keeping the risk prediction model the same as before. Specifically, the measurements of NT‐proBNP are simulated using residuals drawn from an exponential distribution centered around 0 and scaled to the observed variance, and the association parameter relating troponin T and HF hospitalization is assumed to increase over time ($\alpha_{1,2}(t)=0.5+0.1t$).

### 4.2 Results

**FIGURE 2:**
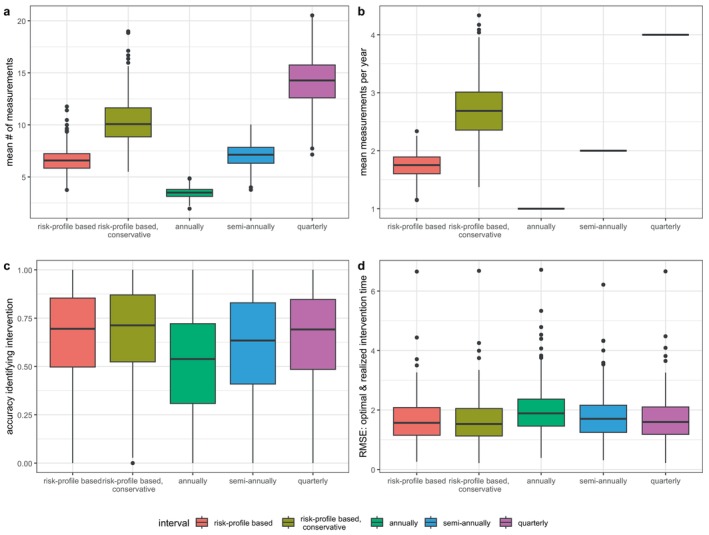
Boxplots comparing the performance of the risk‐based and fixed scheduling strategies, based on 500 simulation iterations.

Figure 2 displays the results of our simulations.

Figure 2a,b compare the number of visits after the warm‐up period between the scheduling strategies. The risk‐based adaptive scheduling strategy requires a median [IQR] of 1.75 [1.60–1.89] measurements per patient year, and the conservative version of this strategy that utilizes the upper limit of the 95% confidence interval of the risk estimates 2.69 [2.36–3.01]. While the annual, semi‐annual and quarterly schedules have 1, 2, and 4 measurements per year by design.

Figure 2c compares the accuracy of the strategies in identifying if interrupting the monitoring process for therapy adjustment is necessary. Here, a patient in need of an interruption is defined as a patient where the optimal time of monitoring interruption falls earlier than their endpoint. The median [IQR] accuracy rate for annual measurements is the lowest with 53.8 [30.8–72.1]%, followed by the semi‐annual schedule 63.4 [40.9–82.9]%. While conservative risk‐based schedules, regular risk‐based schedules, and quarterly fixed schedules perform best with 71.3 [52.4–87.0]%, 69.5 [49.7–85.4]%, and 69.1 [48.5–84.7]%. Figure 2d compares the accuracy of the simulated interruption time to the optimal time of interruption. Again, the fixed strategies performed worse in order of frequency (annual: 1.89 [1.45–2.37], semi‐annual: 1.70 [1.25–2.16], quarterly: 1.59 [1.18–2.10]), while the adaptive strategies performed better (regular risk‐based: 1.56 [1.15–2.08], conservative risk‐based: 1.53 [1.12–2.04]).

Taking both aspects into account, the risk profile‐based strategy achieves a similar accuracy in identifying the need for therapy adjustment to the quarterly schedule while requiring only 1.75 [1.60–1.89] measurements per year. This is in line with prior simulation studies in a smaller subset of the Bio‐SHiFT data set with one (different) endpoint and one biomarker, which also reported efficiency gains for their personalized scheduling strategy compared to fixed scheduling [3, 4]. Using the more conservative version of the risk‐based strategy seems to marginally improve accuracy at the cost of 1 extra measurement per patient year.

**FIGURE 3:**
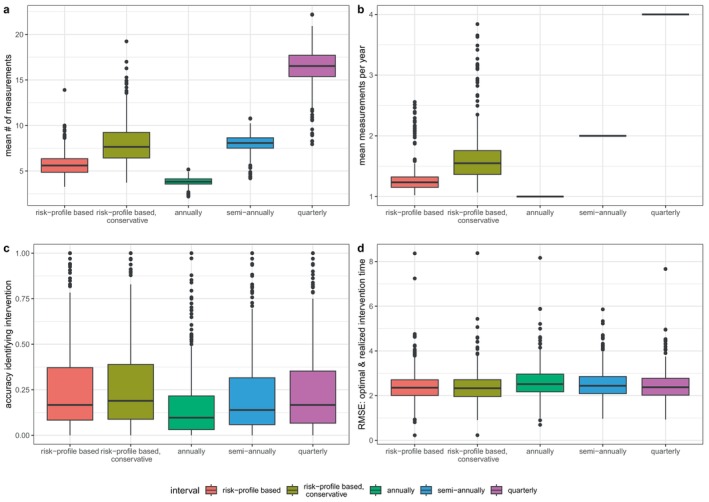
Boxplots comparing the performance of the risk‐based and fixed scheduling strategies, while the joint model is misspecified through violations of the proportional hazard assumption, and the Gaussian assumption for the error terms in the longitudinal part. Based on 500 simulation iterations.

Figure 3 compares the performance of the strategies under model misspecification. In this scenario, the overall accuracy in identifying shifts in risk reduces sharply for all strategies due to the increased prediction error introduced by misspecifying the model (regular risk‐based: 16.7 [8.3–37.1]%, conservative risk‐based: 18.9 [8.8–38.9]%, annual: 9.7 [3.1–21.6]%, semi‐annual: 13.9 [5.9–31.6]%, quarterly: 16.7 [6.7–35.3]%). However, we see a similar pattern as before, where risk‐based strategies perform similar to the quarterly schedules, while using less measurements (regular risk‐based: 1.23 [1.15–1.32], conservative risk‐based: 1.55 [1.36–1.76]). This indicates that even when the joint model is not fully optimized for the data at hand, the risk predictions it provides can still improve the scheduling process.

## 5 Discussion

In this article, we proposed a risk profile‐based scheduling strategy for the monitoring of conditions that use multiple longitudinal biomarkers and are subject to competing events, such as stable CHF patients, and compared the performance of this approach with traditional fixed schedule alternatives via a simulation study. Our results show that the risk profile‐based scheduling approach has clear advantages over monitoring visits on a regular schedule. Its accuracy in identifying the risk of a patient exceeding our threshold Δ rivals that of quarterly measurements while reducing the number of visits per patient year by 56.2%. As such, every measurement seems to be used more efficiently. Moreover, the sensitivity analysis indicates that the adaptive strategy can still outperform fixed schedules when the joint model is misspecified.

We have chosen to make dynamic risk predictions based on joint models for longitudinal and time‐to‐event data; however, as it only depends on the risk estimate, any model capable of making these predictions, such as landmarking or machine learning‐based approaches, could be used in this strategy. The model used should be picked carefully. While our study highlights the benefits of these adaptive scheduling methods, poorly calibrated prediction models could potentially lead to worse monitoring performance. Further limitations of our study include that we do not account for the fact that parameters that trigger therapy adjustments and new measurements could be varied by the clinician on a case‐by‐case basis, which could potentially influence performance. However, previous work on risk‐based scheduling has shown that similar results are robust to smaller changes in these parameters [3, 4].

Risk‐profile based monitoring intervals provide significant benefits in our CHF‐based simulation study. The simulations demonstrate that incorporating personalized risk predictions can outperform fixed scheduling strategies with more frequent biomarker measurements. As chronic conditions that require vigilant monitoring for disease aggravation—such as CHF—become more common, there is a great need for methods that can improve the accuracy and efficiency of monitoring, to reduce the burden on the patient and resource usage. By including competing risks and multiple biomarkers in our scheduling strategy, risk‐based scheduling strategies could become a more realistic option for clinical use. Future research should focus on an open and transparent software implementation to make adoption research into these adaptive schedules more feasible, and could focus on adaptive scheduling strategies tailored to other patient monitoring settings, such as those with multi‐state or recurrent survival models, or where longitudinal measurements are not necessarily taken at the same time.

## Conflicts of Interest

I.K. received travel support from Olink and SomaLogic. The other authors have no conflicts of interest to report.

## Supporting information


**Supplementary Data S1**.

## Data Availability

The data that support the findings of this study are available from the corresponding author upon reasonable request.
